# An innovative immunotherapeutic strategy for ovarian cancer: CLEC10A and glycomimetic peptides

**DOI:** 10.1186/s40425-018-0339-5

**Published:** 2018-04-17

**Authors:** Laura L. Eggink, Katherine F. Roby, Robert Cote, J. Kenneth Hoober

**Affiliations:** 1 Susavion Biosciences, Inc., 1615 W. University Drive, Suite 132, Tempe, AZ 85281 USA; 2 University of Kansas Medical Center, Kansas City, Kansas, USA

**Keywords:** Glycomimetic peptides, Dendritic cells, CLEC10A, ASGPR-1, N-Acetylgalactosamine, Peritoneal cells, Ovarian cancer

## Abstract

**Background:**

Receptors specific for the sugar *N*-acetylgalactosamine (GalNAc) include the human type II, C-type lectin receptor macrophage galactose-type lectin/C-type lectin receptor family member 10A (MGL/CLEC10A/CD301) that is expressed prominently by human peripheral immature dendritic cells, dendritic cells in the skin, alternatively-activated (M2a) macrophages, and to lesser extents by several other types of tissues. CLEC10A is an endocytic receptor on antigen-presenting cells and has been proposed to play an important role in maturation of dendritic cells and initiation of an immune response. In this study, we asked whether a peptide that binds in the GalNAc-binding site of CLEC10A would serve as an effective tool to activate an immune response against ovarian cancer.

**Methods:**

A 12-mer sequence emerged from a screen of a phage display library with a GalNAc-specific lectin. The peptide, designated svL4, and a shorter peptide consisting of the C-terminal 6 amino acids, designated sv6D, were synthesized as tetravalent structures based on a tri-lysine core. In silico and in vitro binding assays were developed to evaluate binding of the peptides to GalNAc-specific receptors. Endotoxin-negative peptide solutions were administered by subcutaneous injection and biological activity of the peptides was determined by secretion of cytokines and the response of peritoneal immune cells in mice. Anti-cancer activity was studied in a murine model of ovarian cancer.

**Results:**

The peptides bound to recombinant human CLEC10A with high avidity, with half-maximal binding in the low nanomolar range. Binding to the receptor was Ca^2+^-dependent. Subcutaneous injection of low doses of peptides into mice on alternate days resulted in several-fold expansion of populations of mature immune cells within the peritoneal cavity. Peptide sv6D effectively suppressed development of ascites in a murine ovarian cancer model as a monotherapy and in combination with the chemotherapeutic drug paclitaxel or the immunotherapeutic antibody against the receptor PD-1. Toxicity, including antigenicity and release of cytotoxic levels of cytokines, was not observed.

**Conclusion:**

sv6D is a functional ligand for CLEC10A and induces maturation of immune cells in the peritoneal cavity. The peptide caused a highly significant extension of survival of mice with implanted ovarian cancer cells with a favorable toxicity and non-antigenic profile.

**Electronic supplementary material:**

The online version of this article (10.1186/s40425-018-0339-5) contains supplementary material, which is available to authorized users.

## Background

CLEC10A (Ca^2+^-dependent lectin-type receptor family member 10A, CD301) is an endocytic receptor that has been proposed as a target for immunotherapy of cancer [1–3]. C-Type lectin receptors require Ca^2+^ for binding the sugar ligand, both to achieve correct structure of the binding site and coordination with sugar hydroxyl groups [4–7]. CLEC10A (also designated the macrophage galactose-type lectin, MGL) is expressed on dermal dendritic cells, immature peripheral dendritic cells, alternatively-activated M2a macrophages, and other tissues [1, 2, 8–11]. The receptor constitutively initiates a Ca^2+^ signal upon ligand-induced endocytosis [12]. Ca^2+^ and the ligand dissociate from the receptor in early endosomes; the ligand is processed through the MHC class I and II pathways for presentation to T cells while Ca^2+^ is transferred to the cytosol and the receptor is recycled [12, 13]. An elevation of cytosolic Ca^2+^ is the ubiquitous second messenger [14, 15] involved in stimulation of maturation and subsequent functions of dendritic cells (DCs) [16, 17].

CLEC10A is a pathogen-recognition receptor that is highly specific for structures that contain terminal *N-*acetylgalactosamine (GalNAc). Endogenous examples of these structures include the blood group A substance (GalNAcα1,3[Fucα1,2]Gal-), in which GalNAc and fucose are linked to the penultimate residue, galactose (Gal) [18]. These structures tend to have low affinity for the receptor. Nevertheless, terminal glycans are often antigenic, and individuals with each blood type express antibodies against the other. The first step in *O*-glycosylation of cell membrane-bound and secreted glycoproteins occurs in the Golgi by a family of up to twenty distinct UDP-*N*-acetyl-α-D-galactosamine:polypeptide *N*-acetylgalactosaminyltransferases (GALNTs), which attach GalNAc to the hydroxyl group of serine (Ser) or threonine (Thr) [19]. This reaction is essential for the synthesis of larger *O-*glycans and the production of mucins, which are major glycoproteins engaged in maintenance of epithelial tissues [20]. An almost universal feature of cancer cells is the expression of an aborted *O*-glycan, in which only GalNAc is attached to Ser/Thr, a structure known as the Tn antigen [21]. A total of 96 glycoproteins bearing one or more Tn antigens was identified on human T lymphoblastoid cells (Jurkat cell line) and 33 glycoproteins were identified on human breast adenocarcinoma cells (MCF7 cell line) [22]. Antigenicity of the Tn structure was demonstrated in the mouse, and the induced antibodies provided protection against an implanted tumor cell line [23, 24]. Addition of multiple Tn antigens to mucin-derived peptides enhanced immunogenicity and promoted the use of these glycopeptides as vaccines [24–28]. Tumor cells also express the TF antigen (Galβ1-3GalNAcα1-*O*-Ser/Thr) [21, 29]. Whereas the appearance of Tn and TF antigens have been considered the result of incomplete assembly of the typical tri- or tetra-saccharide *O*-linked glycans, recent evidence suggests that a shift in regulatory pathways driven by membrane trafficking events leads to the shorter glycans on cancer cells [30].

Efficient engagement of CLEC10A requires multivalent ligands such as a fragment of the MUC1 protein bearing 9 Tn moieties [2, 28, 31] or multimeric Tn-peptide structures [24, 32] that provide orders of magnitude greater avidity to the receptor than a single GalNAc residue. An immune response is initiated by internalization of the ligand by trimers of CLEC10A on DCs [33], antigen processing, migration over several days to draining lymph nodes, and subsequent presentation of the antigen to naïve, antigen-specific T cells [34–36]. The structure of the ligand influences the cellular response, with large Tn-bearing glycoproteins trapped in an endolysosomal compartment [28] whereas smaller glycopeptides are further processed in HLAI/HLAII compartments [2, 28].

The type II C-type lectin asialoglycoprotein receptor-1 (ASGPR-1, Ashwell-Morell receptor, CLEC4H1) on hepatic cells also binds GalNAc-containing structures [37, 38]. ASGPR-1 has a 60-fold greater preference for GalNAc over Gal [39]. Each healthy rat hepatocyte expresses 4 to 5 × 10^5^ molecules of ASGPR-1 as binding sites for asialo-orosomucoid [40] or 1.8 × 10^6^ molecules as determined by an ASGPR-1-targeted antibody [41], which provides a surface concentration for the receptor of 0.8 to 1 μM. Although Gal is the terminal sugar on the multivalent glycans of asialo-orosomucoid [42], the protein binds ASGPR-1 with a K_D_ of 2 to 7 × 10^− 9^ M [40, 43]. Blood proteins and cells whose bound glycans have lost terminal sialic acid bind ASGPR-1, which internalizes the ligand for degradation [38]. Other major GalNAc/Gal-specific receptors include the type II C-type lectin CLEC4F, which is expressed by Kupffer cells in liver [44]; and the scavenger C-type lectin receptor, with a preference for Gal [45]. The mouse expresses two forms of MGL, MGL1 specific for Gal and MGL2 specific for GalNAc [8]. Human CLEC10A is similar to mouse MGL2, preferentially binds GalNAc, and recognizes terminal GalNAc-containing residues such as the Tn antigen [9].

We asked whether the collective evidence of the role of ligands of CLEC10A, such as promoting DC maturation, T cell activation, and initiation of an immune response would coalesce into an effective anti-cancer therapy. To achieve this goal, the ligand should serve as a ‘trigger’ to initiate a cascade of events that will lead to activation of the immune system [1, 2, 31, 46]. Given that natural ligands of CLEC10A that contain GalNAc bind with low affinity and are antigenic, we considered whether a multivalent peptide mimetic of GalNAc would serve this purpose. We previously described efficacy of a tetravalent, 12-amino acid peptide sequence, svL4, which emerged from a screen of a phage display library [47], in a murine model of a brain glioma in combination with brief radiation [48]. Here we report that a tetravalent structure with the C-terminal 6-mer sequence, sv6D, retains the binding activity of svL4 and is a more potent stimulator of immune cells. The peptides bind with high avidity to lectins specific for GalNAc. Analysis of the sequences of the peptides with MHC binding databases [49, 50] predicted that they would not likely be presented by MHC class I or MHC class II molecules in humans or induce production of antibodies. Indeed, no antibodies were detected in mouse sera after alternate-day subcutaneous injections over 3 months. Subcutaneous injection of the peptides stimulated proliferation and maturation of immune cells in the peritoneal cavity. Effectiveness as an anti-cancer approach is demonstrated by the inhibition of accumulation of ascites in a murine model of ovarian cancer.

## Methods

### Animals

C57BL/6 and Balb/c mice were obtained from Charles River Laboratories (Wilmington, MA). Studies of the effects of subcutaneous injection of peptides on populations of peritoneal cells were performed in AAALAC-accredited facilities at Biomodels LLC, Watertown, MA (IACUC approval number 13-0611-01). Studies with the murine model of ovarian cancer were conducted at the University of Kansas Medical Center, Kansas City, KS (IACUC approval number 2015-2288). Cells of an ovarian epithelial cancer line, ID8 [51], were implanted into the peritoneal cavity of female C57BL/6 mice at a dose of 6 X 10^6^ cells. All animals were weighed at least weekly throughout these studies. Accumulation of ascites was monitored by increase in body weight, and animals were euthanized when end-stage behavior was expressed. No animals were euthanized for any reason unrelated to the cancer prior to termination of the experiment. Kaplan-Meier survival curves were analyzed by the Mantel-Cox log-rank test.

### Synthesis of peptides

The sequence of svL4 was identified by screening a 12-mer phage display library (New England BioLabs, Ipswich, MA) with the lectin from *Helix pomatia*, which is specific for GalNAc [47, 52]. The consensus sequence was incorporated into a tetravalent structure based on the concept of avidity of binding to receptors as a function of ligand density and entropic factors [38, 43, 53–55]. Multivalent peptides were synthesized by standard solid-phase chemistry utilizing Fmoc (9-fluorenylmethoxycarbonyl)-protected amino acids by CBL Biopharma LLC (Patras, Greece). The tri-lysine ‘core’ was synthesized on the solid-phase resin and extended with the sequence GGS. The ‘arms’ with C-terminal G were synthesized separately by standard chemistry and condensed in solution with the core [56]. Modifications at the C-terminus consisted of an amide group (no tag) or extensions with ε-biotinyl-lysinyl-amide. The sequence GGGS was included in the structure as a linker between the mimetic sequence and the tri-lysine core. Derivatives of svL4 were also synthesized to determine whether subsets of the sequence have differing activities. Structures, designations and molecular weights of svL4 and derivatives are shown in Table 1.

**Table 1:** Peptides synthesized as subsets of svL4

Sequence	Code	MW
[(VQATQSNQHTPR-GGGS)_2_K]_2_K-NH_2_	svL4	6826
[(VQATQS- GGGS)_2_K]_2_K-NH_2_	svC1	3893
[(VSNQH- GGGS)_2_K]_2_K-NH_2_	svD2	3697
[(NQHTPR-GGGS)_2_K]_2_K-NH_2_	sv6D	4369

### Preparation of endotoxin-negative peptide solutions

The peptides were purified to greater than 95% by HPLC on a XDB-C8 column using a gradient of 5% to 25% acetonitrile in 0.1% trifluoroacetic acid in water at a column temperature of 60 °C. Quality of the synthetic product, including correct synthesis and purity, was assessed by MALDI and ESI mass spectroscopy. Lyophilized peptides were dissolved in 100 mM NaCl, neutralized to pH 5, and adsorbed onto a CM-Sephadex C-25 column (2.5 × 10 cm). The column was washed with 200 mM NaCl to ensure removal of lipopolysaccharide (LPS) prior to elution of svL4 with 500 mM NaCl. LPS was not detected in the peptide solution eluted from the CM-Sephadex column (< 0.01 EU/10 mg peptide). Although sv6D and svL4 have the same molecular charge (+ 8.4 at pH 7.0), elution of sv6D from the CM-Sephadex column required 1 M NaCl. Peak fractions were combined and diluted with endotoxin-negative water (HyClone Laboratories, Inc., Logan UT) to achieve a NaCl concentration of 150 mM. The sample was further diluted to the desired concentration with endotoxin-negative phosphate-buffered saline, pH 7.4 (PBS) (Sigma-Aldrich, St. Louis, MO), and filter-sterilized. The presence of LPS was assayed by a quantitative colorimetric assay with the *Limulus* amebocyte lysate (LAL, Lonza, Walkersville, MD). Concentration of svL4 was determined with the bichinichonic (BCA) assay (Pierce Biotechnology, Inc., Rockland, IL) with the dansylated peptide [47] or bovine serum albumin as standards. Absorbance of analyzed solutions provided an extinction coefficient for svL4 of 22 OD units/mg/mL at 210 nm, which was also used for determination of concentration of sv6D. This value was supported by calculations from absorbance coefficients of peptide bonds in small proteins [57] and was used as a convenient measure of concentration.

### Binding assays

Solid-phase binding assays were performed with streptavidin-, protein A/G-, or Nickel-coated microtiter plates (Pierce). His-tagged or Fc-fusion recombinant receptors (R&D Systems, Minneapolis, MN) were reconstituted in PBS. Sufficient receptors were added to wells to saturate the coating. His-tagged receptors were added to each well at a 5-fold excess over the stated capacity of the Ni coating to minimize non-specific binding of the peptide. Wells were washed three times with binding buffer (25 mM Tris HCl, pH 7.4, 150 mM NaCl, 0.05% Tween-20) to remove unbound receptor and 50 μL of 2 μM biotinylated peptide in binding buffer were added and allowed to incubate for 1 h. The wells were washed four times with binding buffer and then incubated with 50 μL of 1 μg/mL streptavidin conjugated with peroxidase (Sigma-Aldrich) for 1 h. The wells were washed five times with binding buffer and 50 μL of peroxidase substrate (1-Step™ Ultra TMB, Pierce) were added. After several minutes to allow color development, the reaction was stopped with 50 μL 1 M H_3_PO_4_ and absorbance was measured at 450 nm. Bound streptavidin was quantitated by the specific activity of peroxidase (absorbance/ng protein conjugate/min) under the conditions of the assay.

### Cytokine assays

An allogenic mixed leukocyte reaction was performed with 5 × 10^3^ human monocyte-derived DCs co-cultured with 1 × 10^5^ CD3^+^ T cells in X-VIVO 20 medium (Lonza) by Astarte Biologics, Inc. (Bothell, WA). IFN-γ in the medium was assayed over a period of 5 days with a Meso Scale Discovery assay kit (Meso Scale Discovery, Rockville, MD). For analysis of a cytokine response in vivo to subcutaneous injection of peptide, 6 to 8 week-old female Balb/c mice were anesthetized with isoflurane and inoculated with 5 × 10^5^ 4 T1 breast cancer cells in the 4th mammary fat pad. When tumors reached a volume of at least 500 mm^3^, animals were randomized into groups of 3 and dosed with either 0.1 or 1.0 nmole/g of svL4. At 4 h post dose, terminal blood was collected from 3 animals per group and prepared for serum. Sera were also collected from healthy Balb/c mice by the same procedure. Changes in the levels of cytokines/chemokines in sera from breast cancer-bearing and healthy mice were analyzed with the mouse L-308 membrane array by RayBiotech, Inc. (Norcross, GA).

### Flow Cytometry

Healthy C57BL/6 male mice, 10 weeks old, were injected subcutaneously with peptides on alternate days. Peritoneal cells were isolated from mice 24 h after dosing by injecting ice cold PBS (containing 3% FBS) into the peritoneal cavity, gently massaging the abdomen, and then collecting the fluid, which was transferred into K_2_EDTA-treated tubes for evaluation of specific biomarkers. Cells from three mice in each group were pooled, counted and divided among FACS tubes (1 × 10^6^ cells per tube). Cells were washed once in PBS containing 3% FBS, then stained with 0.25 to 1 μg of fluorescently conjugated antibody per 10^6^ cells in 100 μL of buffer as recommended by the manufacturer (Table 2).

**Table 2:** List of cell surface markers used in this study

Antibody	Source	Catalog no.	Cell type marker
CD11b-APC	BioLegend	101,212	Macrophage
CD11c-Pacific Blue	BioLegend	117,322	Myeloid Cell, DC
CD11c-VioBlue	Miltenyi	130-102-797	Myeloid Cell, DC
CD3ε-VioBlue	Miltenyi	130-102-203	T Cell
CD4-APC	BioLegend	100,412	T_H_ Cell
CD8α-PE	BioLegend	100,708	Cytotoxic T Cell
CD19-FITC	BioLegend	115,506	B Cell
CD69-FITC	BioLegend	104,505	Cell Activation
CD73-Brilliant Violet	BioLegend	127,215	B Memory Cell
CD80-APC	BioLegend	104,713	B Cell Activation
CD86-APC	BioLegend	105,011	Cell Activation
CD273-PE	BioLegend	107,205	B Memory Cell
F4/80-PE	BioLegend	123,110	Macrophage
Ly6C-FITC	Miltenyi	130-093-134	Monocyte
NK1.1-APC	Miltenyi	130-095-869	NK Cell

Cells were incubated with the antibodies for 30 min at 4 °C in the dark. Following staining, the cells were washed two times with PBS/3% FBS and immediately analyzed with a MACSQuant flow cytometer (Miltenyi Biotech, Inc., San Diego, CA). Compensation was performed using single stain tubes for each color present in the analysis as well as an unstained control sample. Collection of flow cytometric data and analysis with FlowJo software, version 10.0.6 (FlowJo, LLC, Ashland, OR) were performed by Biomodels LLC (Watertown, MA).

### Antigenicity assay

The sequence of one arm of svL4 or sv6D was conjugated to keyhole limpet hemocyanin (KLH) and injected into rabbits at two week intervals (total of 3 injections) (New England Peptide, Inc., Gardner, MA). Two weeks after the final injection, sera were prepared. Mice were injected with a peptide every other day over a period of 3 months (total ~ 45 injections), blood was then collected by cardiac puncture and sera were prepared. Rabbit serum was diluted 1:10, whereas mouse serum was diluted 1:1 or 1:5 with PBS containing 0.05% Tween-20 (PBST). Sera were incubated in microtiter wells coated with protein A/G (Pierce) for 90 min. The wells were washed three times with PBST, and then incubated with 50 μL of 1 μM biotinylated svL4 or sv6D for 1 h. The wells were washed four times with PBST, incubated 1 h with 1 μg/mL streptavidin-peroxidase conjugate, and washed four times with PBST. Peroxidase activity was assayed as above under Binding Assays.

## Results

### Peptides as mimetics of GalNAc

**Fig. 1:**
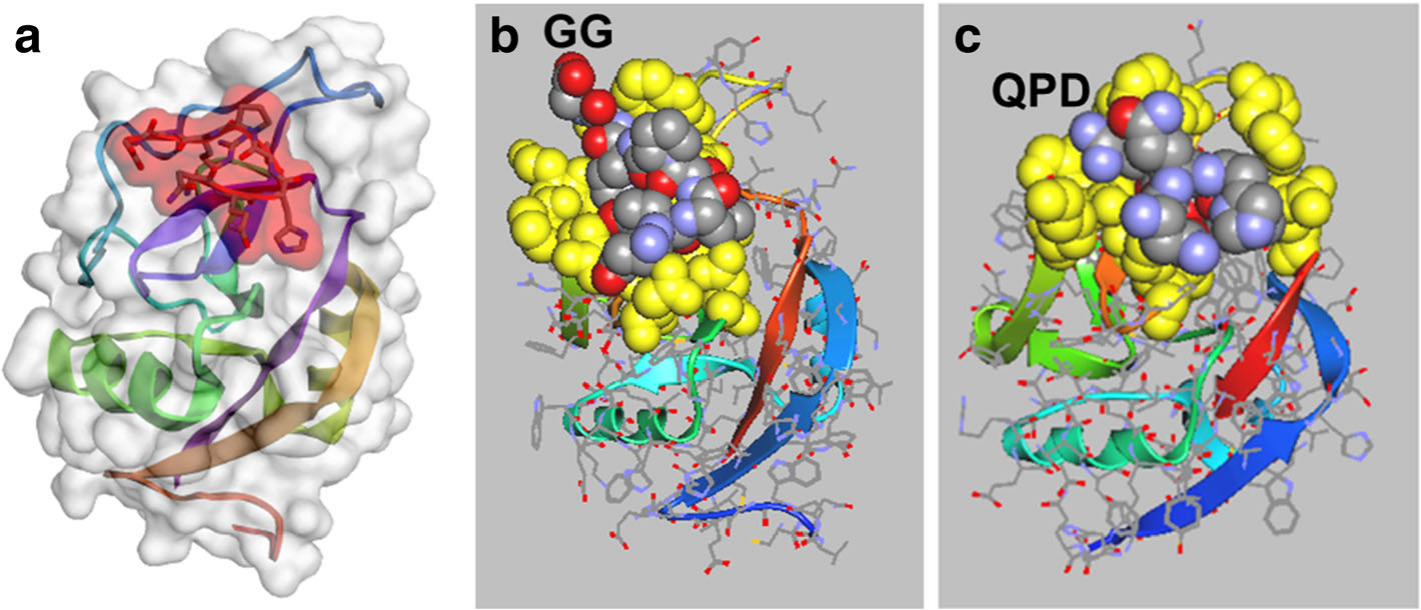
Docking of sv6D to receptors. **a** In silico docking of an arm of sv6D (NQHTPRGG) to the carbohydrate-recognition domain of ASGPR-1 (accession number 1DV8) with CABS-dock (RMSD = 0.7611 Å) [60]. The peptide is enclosed in red shading that delineates the space-filling molecular structure. **b** The structure in (**a**) as rendered in ArgusLab 4.0.1 (predicted binding energy, ΔG’ = − 40 kJ/mol). Amino acids in the binding site that interact with the peptide are colored in yellow as space-filling structures. sv6D is colored as carbon, grey; nitrogen, blue; and oxygen, red. A portion of the linker to the remainder of the tetravalent structure is indicated as GG. **c** The structure of CLEC10A was generated with SWISS-MODEL Deep View [58, 59] from the structure of ASPGR-1. Docking was modeled with CABS-dock (RMSD = 1.421 Å) and downloaded into ArgusLab 4.0.1 (predicted binding energy, ΔG’ = − 38 kJ/mol). The binding site and peptide are colored as in (**b**). The location of the QPD sequence is indicated. Helical and beta-strand secondary structures of the protein are shown as colored ribbons

The functional sequence of svL4 was identified through a screen of a phage display library with the GalNAc-specific lectin from the snail *Helix pomatia* [47]. To investigate further its ability to bind to human GalNAc-specific lectins, in silico molecular modeling was performed. Whereas the crystal structure of CLEC10A has not been reported, that of ASGPR-1 (CLEC4H1) was determined [7]. The extensive homology between CLEC10A and ASGPR-1 allowed generation of a likely structure for CLEC10A with SWISS-MODEL Deep View [58, 59]. The CABS-dock modeling program [60] was used to predict whether the peptide would bind to ASGPR-1 and CLEC10A. This method searches for a binding site on the protein without prior assignment and defines the most probable peptide conformation. The docking program accommodated svL4 in the carbohydrate-recognition domain (CRD) in a hair-pin conformation (RMSD = 0.868 Å) (not shown). As shown in Fig. 1a, the shorter sv6D, with a length dimension approximately two-times that of a sugar residue, was accommodated within the CRD of ASGPR-1. A portion of the linker was included in the peptide (i.e., NQHTPRGG) to indicate the orientation of the peptide and, by extension, the remainder of the tetravalent peptide. The GG sequence is more clearly seen after energy minimization in the model rendered by ArgusLab in Fig. 1b. Similar binding predictions were obtained with pepATTRACT [61] and MDockPeP [62], which are blind docking programs that allow the fully flexible peptide to adapt to surface properties of the proteins [63, 64]. ASGPR-1, and by homology CLEC10A, has an acidic GalNAc binding site, which may contribute through electrostatic interaction to binding avidity of the positively-charged peptide. The docking programs orient the peptide with the Arg (R) residue near the sequence Gln-Pro-Asp (QPD) that specifies binding of C-type lectins to GalNAc residues (indicated in Fig. 1c) [65, 66]. In silico replacement of QPD with Glu-Pro-Asn (EPN), the sequence that has been described as a determinant for binding of mannose [65, 66], as occurs in the mannose receptor and DC-SIGN (dendritic cell-specific intercellular adhesion molecule (ICAM)-3 grabbing non-integrin, CD209) [67], did not significantly alter the predicted binding of sv6D to CLEC10A. The flexibility of the peptide likely allows interaction of the Arg residue with negatively-charged Asp or the nearby Glu. As a further test, the CABS-dock program predicted much weaker binding of sv6D to DC-SIGN (RMSD = 4.477 to 7.405 Å compared with 0.7611 to 1.421 Å for ASGPR-1 and CLEC10A) and with significantly lower predicted binding energy (not shown). The lack of binding to DC-SIGN, as described below, apparently results from the low homology of the amino acid sequences of the receptors within the CRD. These results suggest that specificity of peptide binding in the CRD is largely provided by the properties of the protein surface, as is typical for most peptide-protein interactions [63]. Although the conformation of the CRD of the GalNAc-specific lectin from *Helix pomatia* is similar to that of C-type lectins [52], the snail lectin lacks a QPD sequence or significant homology to CLEC10A. However, the CABS-dock program indicated an RMSD = 1.604 Å for binding of sv6D, with predicted binding energy of ΔG’ = − 38 kJ/mol (not shown).

### Binding to recombinant receptors

Solid-phase binding assays were performed with recombinant human receptors. Three variations of the binding assay were used in this study, all of which allowed the arms of the tetravalent peptide to have full flexibility. (i) Biotinylated peptide was bound in microtiter wells coated with streptavidin and incubated with recombinant human receptors. After extensive washing, the bound receptor was measured by peroxidase conjugated to a receptor-specific antibody. (ii) The extracellular domains of receptors, which were fused with the Fc domain of immunoglobulin IgG, were bound in wells coated with protein A/G. Biotinylated peptide was incubated with the receptors and, after extensive washing, bound peptide was measured with streptavidin conjugated with peroxidase. Or (iii), recombinant receptors with a poly-His tag were bound to Nickel-coated wells and incubated with biotinylated peptide. svL4 and sv6D contain His residues (see Table 1) and can bind to the Ni coating, which was minimized by adding sufficient poly-His-tagged receptor to saturate the Ni coating.

**Fig. 2:**
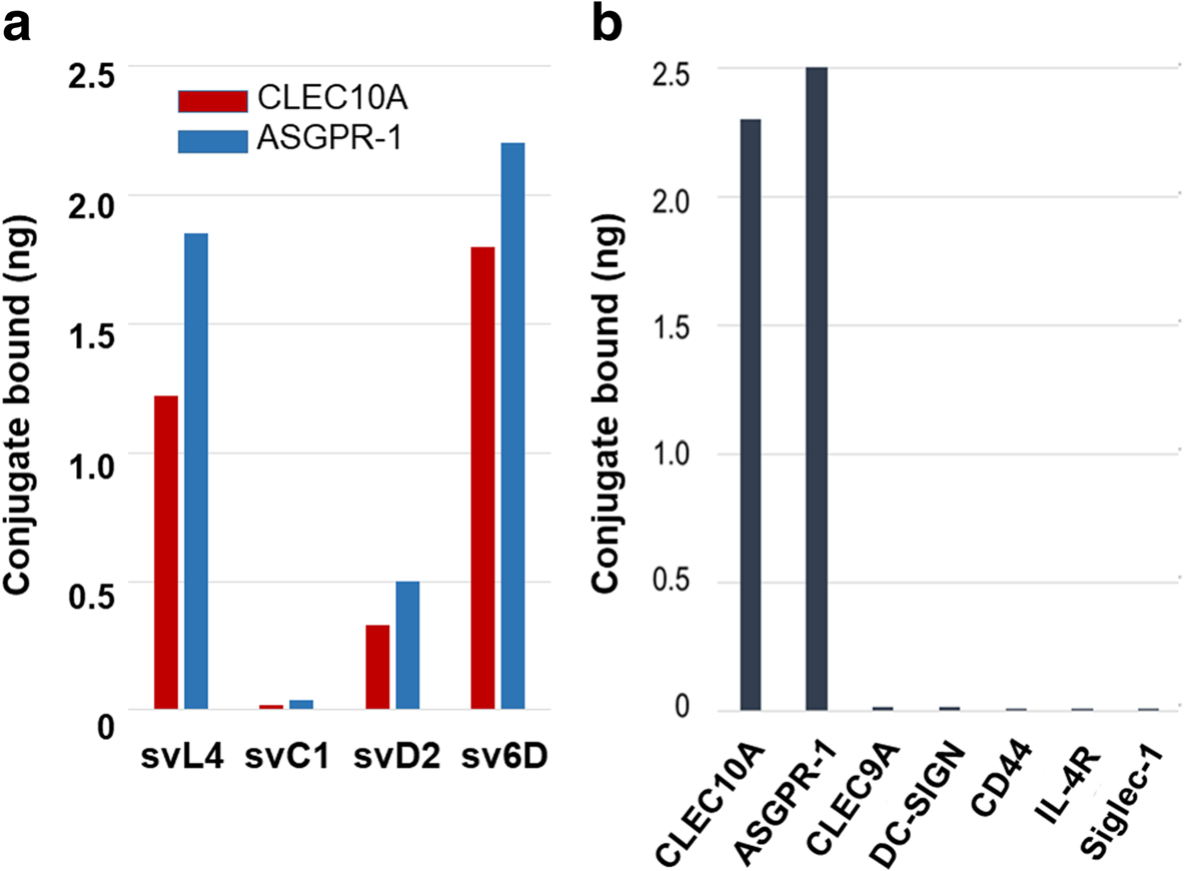
Binding activity of svL4 and sv6D to CLEC10A and ASGPR1. **a** Solid-phase binding assays of subsets of svL4 were performed with recombinant human CLEC10A (red) or ASGPR-1 (blue) as described in Materials and Methods. The peptide structures are shown in Table 1. The figure shows representative data from 3 independent experiments. **b** Binding of sv6D to recombinant human receptors. Extent of binding was corrected for the masses of the recombinant proteins, assuming a single binding site per subunit. The data shown are representative of the results of 4 independent assays

In these assays, svL4 bound to CLEC10A and, as confirmation of GalNAc mimicry, also to ASGPR-1 (Fig. 2). Although a significant consensus was found for the 12-mer sequence of svL4 in the screen of a phage display library with the GalNAc-specific lectin [47], subsets of the sequence were synthesized as shown in Table 1 to test the activity of the N-terminal and C-terminal halves in comparison with the full-length svL4. As shown in Fig. 2a, the C-terminal half (sv6D) bound as strongly as the full-length sequence to CLEC10A and to ASGPR-1. Retention of the N-terminal half (svC1) was near the level of blank wells. The mid-section of the svL4 sequence (svD2) bound weakly to these receptors.

A survey of other receptors showed that svL4 or sv6D did not bind significantly to CLEC9A, a C-type lectin receptor expressed on monocytes and DCs [68]; DC-SIGN, a mannose-specific lectin-type receptor [67, 69, 70]; or Siglec-1, which is specific for terminal Neu5Ac-Gal/GalNAc-sequences on complex glycans [71, 72]. Binding was not detected with CD44, a receptor for hyaluronic acid [73], or IL-4R, a receptor for IL-4 (Fig. 2b). Of the receptors we have assayed thus far, the peptides bound to those specific for GalNAc, which included CLEC4F, a GalNAc-binding receptor expressed by Kupffer cells that is also homologous to ASGPR-1 [44].

**Fig. 3:**
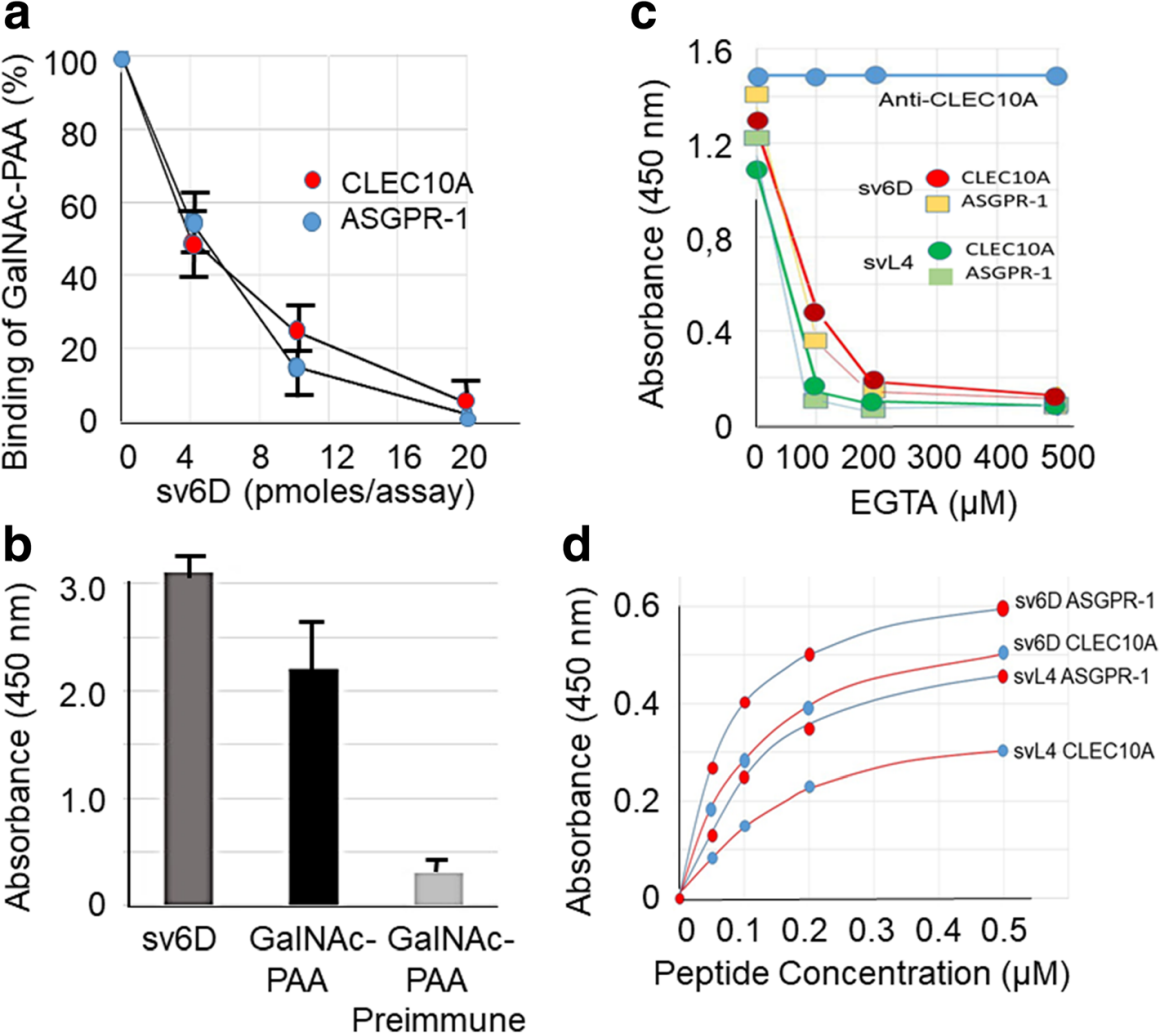
sv6D as a mimetic of GalNAc. **a** Inhibition by sv6D of binding of multivalent GalNAc-PAA (GlycoTech Corp., Frederick, MD) to recombinant rat CLEC10A and human ASGPR-1. The reaction mixture included approximately 200 pmoles of biotinylated GalNc-PAA and increasing concentrations of peptide. The figure includes values ± S.D. from 3 independent experiments. **b** Biotinylated sv6D or GalNAc-PAA were incubated with rabbit antiserum raised against the 6D sequence (NQHTPR) conjugated to KLH. Binding was detected with streptavidin-peroxidase conjugate. Similar data were obtained in 2 experiments. **c** Inhibition by EGTA of binding of sv6D (red, yellow) or svL4 (dark green, light green) to CLEC10A and ASGPR-1, respectively. EGTA (1 mM) was added to the final concentrations indicated to assays. Retention of bound CLEC10A was determined by incubation with biotinylated anti-CLEC10A (goat IgG, R&D Systems) and detection with streptavidin-peroxidase conjugate (blue). Similar data were obtained in 3 experiments. **d** Binding of svL4 and sv6D to human recombinant CLEC10A and ASGPR-1 as a function of concentration of peptide in the assay. The figure is representative of 4 separate assays. K_D_ values ± S.D. from reciprocal plots of these data are provided in the text

To further determine whether sv6D interacts with the actual sugar binding site, the ability of sv6D to compete with GalNAc-PAA for binding was assayed. As shown in Fig. 3a, sv6D inhibited binding of GalNAc-PAA to CLEC10A from rat as well as human ASGPR-1. This experiment suggests that the peptide has an avidity over an order of magnitude greater than that of the multivalent GalNAc-PAA. Further support for the mimicry of sv6D was the finding that an antiserum that was raised in rabbits against the sv6D sequence (NQHTPR) conjugated to KLH also bound GalNAc-PAA (Fig. 3b).

Binding of sugar ligands to C-type lectins requires Ca^2+^ bound at three or four sites in the protein [5–7, 67]. Removal of Ca^2+^ from these sites relaxes the structure of the binding site and causes loss of sugar-binding activity without significant change in the remainder of the protein. Thus we reasoned that whether the peptides bind to these receptors at the sugar-binding site could be ascertained by chelation of Ca^2+^. Indeed, binding of the peptides was completely inhibited by chelation of Ca^2+^ with low concentrations of EGTA (Fig. 3c).

Double-reciprocal plots of binding curves with CLEC10A or ASGPR-1 vs. concentration (Fig. 3d) provided K_D_ values of 0.15 ± 0.02 μM and 0.12 ± 0.01 μM, respectively, for sv6D. Corresponding values for binding of svL4 to CLEC10A and ASGPR-1 of 0.24 ± 0.04 μM and 0.21 ± 0.03 μM, respectively, were obtained. Although multivalency dramatically increases avidity by decreasing the k_off_ rate of binding to receptors [74, 75], it has a finite value, which suggests that the equilibrium K_D_ may be slightly elevated because of the extensive wash steps in the assay. Asialofetuin, a multi-glycosylated protein with an IC_50_ = 45.6 ± 2.7 μM for ASGPR-1 [43], at 75 μM inhibited binding of svL4 (0.2 μM) to ASGPR-1 by 53%, which is consistent with the determined K_D_ value.

### Activation of DCs and T cells with peptide

**Fig. 4:**
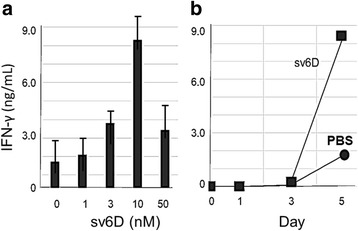
Mixed leukocyte reaction. An allogenic mixture of 5 × 10^3^ human monocyte-derived immature dendritic cells and 1 × 10^5^ negatively selected CD3^+^ T cells were incubated with various concentrations of sv6D. **a** Effect of concentration of sv6D in the medium on release of IFN-γ after 5 days of culture. The figure shows results from an experiment with 3 replicates analyzed by one-way ANOVA, which at 10 nM sv6D, ρ = 0.0064. **b** The time course of the appearance of IFN-γ in the medium of cultures incubated with 10 nM sv6D in (**a**)

Binding of a peptide to CLEC10A expressed by DCs is expected to stimulate maturation and potential activation of T cells [12]. This hypothesis was tested by an experiment in which human monocyte-derived DCs were co-cultured with T cells. Expression of CLEC10A (CD301) by the DCs was established by flow cytometry (data not shown). sv6D was added to the medium at various concentrations, and IFN-γ production by T cells was assayed. As shown in Fig. 4a, the maximal amount of IFN-γ was produced with 10 nM sv6D. Significant release of IFN-γ occurred after 3 days of incubation (Fig. 4b), a time required for DC maturation. These results suggest that the optimal concentration of sv6D for an effect on cellular activity is an order of magnitude lower than the K_D_ obtained from chemical binding assays as in Fig. 3d.

### Responses of immune cells in the peritoneal cavity of healthy mice

To explore their physiological activity in vivo, the peptides were injected subcutaneously into mice. Minimal changes in cell populations were observed in the blood of healthy animals. In contrast, an analysis of total cells in a peritoneal lavage revealed that Balb/c mice contained a large population of small cells (low FSc) with minimal intracellular complexity (low SSc), whereas this population of cells was minimal in C57BL/6 mice (Additional file 1: Figure S1). These observations confirm the results of Festing et al. [76] that peritoneal cells in Balb/c mice expressed a high ‘lymphocyte’ to ‘macrophage’ ratio whereas the reverse occurs in C57BL strains. Within 24 h after injection of peptide, the population of small cells largely disappeared in Balb/c mice but increased in C57BL/6 mice (Additional file 1: Figure S1). Attempts to identify these cells suggested that they comprise a myeloid progenitor population whose proliferation is highly responsive to treatment with the peptide (Additional file 2: Figure S2).

**Fig. 5:**
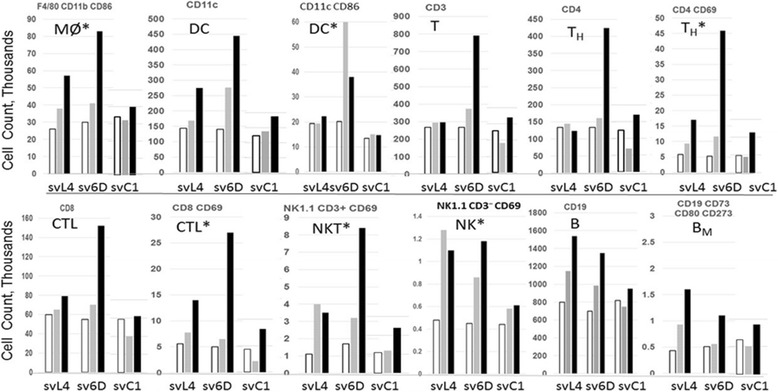
Expansion of peritoneal cells in C57BL/6 mice. Mice were injected subcutaneously at a dose of 1 nmole/g body weight of svL4, sv6D or svC1. Cells in each population recovered in a peritoneal lavage are expressed as cell counts (in thousands). Cells from three mice in each treatment group were pooled for analysis. The number of cells in each population was normalized to the average recovery of total cells from all groups. For each cell type, the left-hand, middle and right-hand set of bars represent samples from mice treated with svL4, sv6D or svC1, respectively. Mice were injected on days 0, 2 and 4, and cells were collected 24 h after each injection. Day 1, open bar; day 3, grey bar; day 5, black bar. Populations that express activation markers are indicated by an asterisk

To examine whether increases occurred in mature immune cells, peritoneal cells from Balb/c and C57BL/6 mice were analyzed by flow cytometry after cells were stained with the markers listed in Table 2. Several-fold increases in macrophages, DCs, T cells and natural killer (NK) cells were found 24 h after a single injection of svL4 (1 nmole/g) into Balb/c mice but a significant increase occurred only after the second injection into C57BL/6 mice. To explore this observation more extensively, svL4 or sv6D was injected into C57BL/6 mice on day 0, 2 or 4. Peritoneal cells were examined by flow cytometry 24 h after each injection. Shown in Fig. 5 are results expressed as cell counts for F4/80 CD11b CD86 (mature, active macrophages); CD11c (DCs); CD11c CD 86 (activated DCs); CD4 CD69 (activated T cells); CD8 CD69 (activated cytotoxic T cells); NK1.1 CD3^+^ CD69 (activated NKT cells); NK1.1 CD3^−^ CD69 (activated NK cells); CD19 (B cells); and CD19 CD73 CD80 CD273 (memory B cells). The numbers of these mature, activated cells continued to increase with each injection. Overall, the responses to sv6D were greater than those to svL4, with large increases in DCs (CD11c^+^ and CD11c^+^ CD86^+^), T cells (CD3^+^, CD4^+^ and CD8^+^) and NKT cells (NK1.1, CD3^+^), although CD19^+^ B cells responded more strongly to svL4. The mean fluorescence intensity did not change significantly, which suggested that the peptides promoted an expansion of mature cell populations. As a control, the expansion of peritoneal cell populations induced by svL4 and sv6D was compared with that of svC1, which as shown in Fig. 2a does not bind to CLEC10A. Populations of specific cell types from animals treated with svC1 did not increase significantly over the 5-day period.

To determine whether the target(s) for svL4 is located on lymphocytes, with subsequent cross-talk signaling to achieve activation of monocytes, an experiment was performed in which svL4 was injected into RAG^—/—^ mice, which lack activity of *rag,* the recombination activation gene. The ability to perform recombination to generate antigen-specific antibodies by V(D)J recombination is required for B and T cell precursors to produce functional antigen receptors on their surface, and without a functional *rag* gene these cells undergo apoptosis [77]. The response of monocytes in the peritoneal cavity of RAG^—/—^ mice to treatment with svL4 was essentially the same as that in C57BL/6 mice (data not shown). The deficiency of B and T cells did not reduce the maturation of monocytes, which suggested that svL4 acts on targets within the myeloid lineage.

### Efficacy of peptides in murine model of ovarian cancer

The strong responses of the peritoneal immune cells to the peptides led us to test whether they would be effective in treating cancers of peritoneal organs. A murine ovarian cancer cell line (ID8) was implanted into the peritoneal cavity of C57BL/6 female mice on day 0. In this system, tumor progression in the peritoneal cavity is initially slow but then progresses rapidly, with ultimate accumulation of ascites [51]. Treatment with sv6D routinely began 45 days after implantation when macroscopic tumor seeds were present, which is analogous to the stage at which most women are diagnosed [78, 79]. Progression of the cancer was monitored by body weight of animals as an indication of ascites accumulation.

**Fig. 6:**
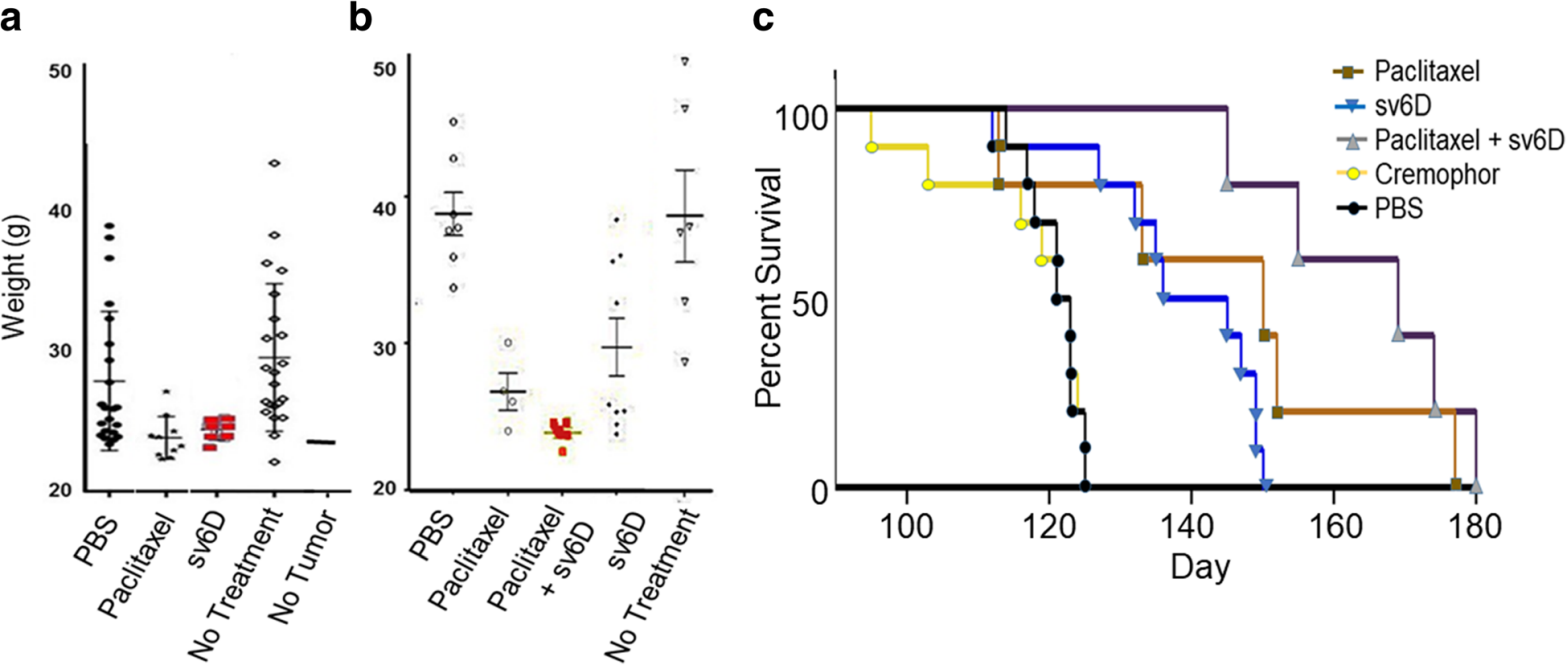
Combination study of sv6D and paclitaxel. **a** Female C57BL/6 mice were implanted with ID8 ovarian cancer cell line. In this experiment, progression of disease was aggressive, with a median survival of 65 days. Body weights of mice on day 58, after two weeks of alternate-day subcutaneous treatment with sv6D (0.1 nmole/g) or 3 intraperitoneal doses (days 45, 47 and 49) of 18 μg/g paclitaxel in cremophor. Analysis of these data by t test with Welch’s correction for unequal variance when applicable indicates *ρ* = < 0.05 for sv6D vs. PBS or no treatment and *ρ* = < 0.05 for paclitaxel vs PBS or no treatment. There was no statistical difference between the effects of paclitaxel and sv6D. **b** Alternate-day injections of sv6D were initiated on day 50 with peptide alone or on day 100 to mice that were previously treated with paclitaxel between day 45 and 49. The figure shows weights of mice on day 121. Analysis of the data set by t test indicated that paclitaxel vs. PBs *ρ* = < 0.05; paclitaxel vs. sv6D was not different, and paclitaxel vs. paclitaxel plus sv6D *ρ* = < 0.05. **c** Kaplan-Meier survival curves for mice treated with paclitaxel, sv6D or a combination of the two in which alternate-day subcutaneous injections of sv6D was started 50 days after paclitaxel treatment. Mantel-Cox log-rank test, *p*_(sv6D vs. PBS)_ = < 0.0001. Paclitaxel, brown; sv6D from day 45, blue; paclitaxel plus sv6D from day 100, purple; cremophor, yellow; PBS, black

Efficacy of sv6D was compared with that of paclitaxel, a standard-of-care chemotherapeutic drug that stabilizes microtubules, interferes with the function of kinetochores, and arrests cells at the G2/M boundary of mitosis [80, 81]. In the experiment illustrated in Fig. 6a, progression of disease was aggressive and evidence of ascites accumulation was observed already at day 45, the time treatment was initiated. After two weeks of treatment, when disease in several control mice had already progressed to end stage, weights of mice treated with sv6D at a dose of 0.1 nmole/g body weight or paclitaxel had not significantly increased. This observation suggested that treatment with sv6D suppressed accumulation of ascites when first evident.

We then asked whether sv6D would be effective *in combination* with paclitaxel. In this study the disease progressed more slowly, with the median end-stage at day 122. sv6D again suppressed accumulation of ascites when treatment was initiated at day 45, with no significant weight gain for half the animals at day 120 (Fig. 6b). Efficacy of sv6D at a dose of 0.1 nmole/g was comparable to that of paclitaxel, with median survival of approximately 141 days (Fig. 6c). Because cancer cells eventually escape paclitaxel treatment, these results led to initiation of treatment with sv6D at day 100, 50 days after treatment with paclitaxel, when accumulation of ascites was first observed in this group. Over the next 3 weeks, alternate-day injections of sv6D completely suppressed further increases in weight of the animals (Fig. 6b). Continued treatment with sv6D resulted in dramatic extension of survival of the animals to a median of 169 days (Fig. 6c).

### Efficacy of sv6D in combination with anti-PD-1

**Fig. 7:**
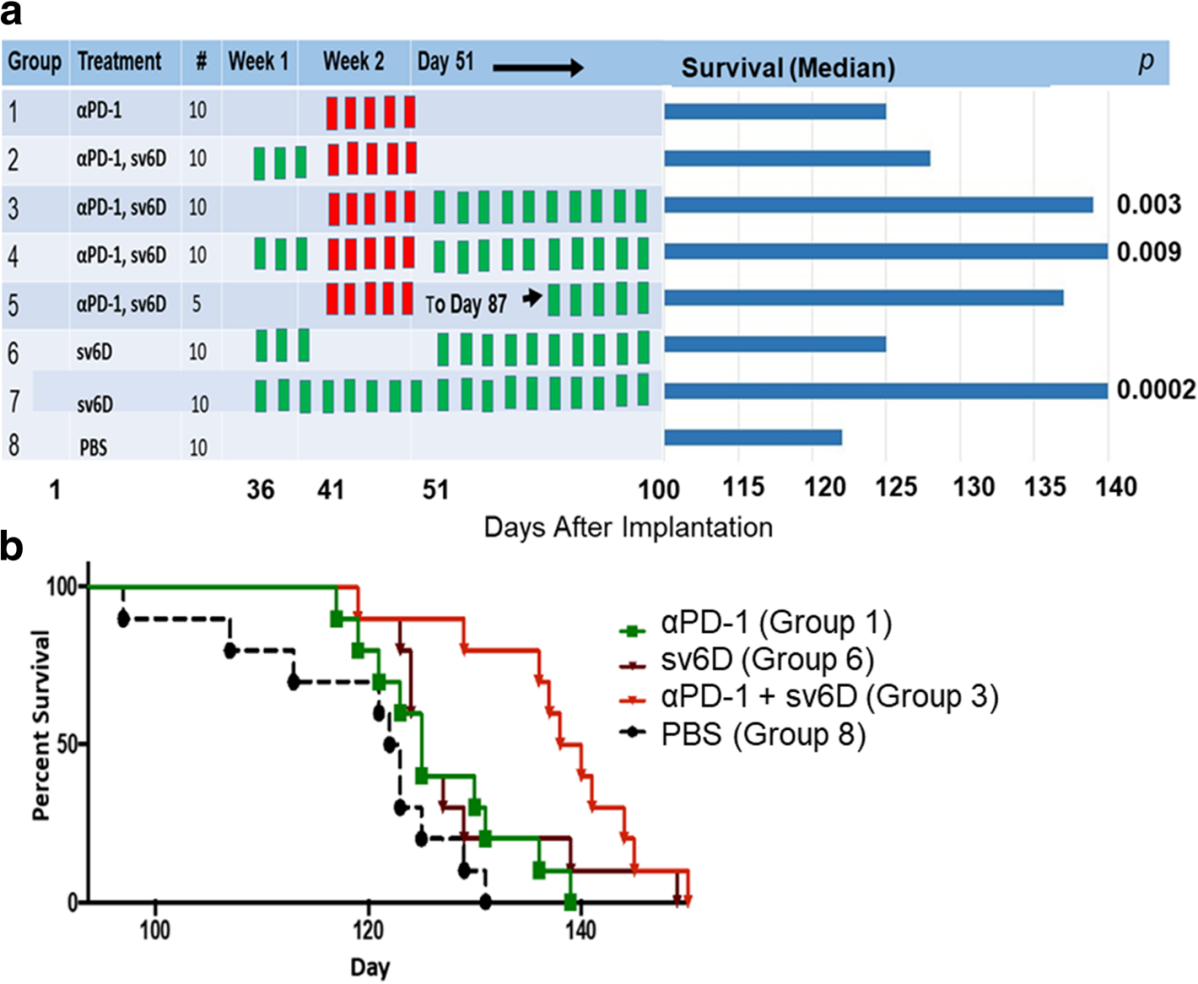
Combination study of sv6D and anti-PD-1. **a** The design of the experiment and extension of survival of C57BL/6 female mice implanted with ovarian cancer cell line ID8 and treatments with anti-PD-1 and sv6D. Anti-PD-1 (rat anti-mouse, clone 29F.1A12) was administered intraperitoneally every other day, 200 μg protein per dose, between day 41 and 49 (red bars). sv6D was administered subcutaneously every other day at 0.1 nmole/g, starting on day 36, 51 or 87 (green bars). Survival data were analyzed by the Mantel-Cox log-rank test to determine ρ values. **b** Kaplan-Meier survival curves of mice treated with anti-PD-1 and/or sv6D. The figure shows survival of groups 1, 3, 6 and 8. For group 3, the Mantel-Cox log-rank test *p* = 0.003 indicates the significance between treatment with the combination and antibody alone. Anti-PD-1, green; sv6D, brown; anti-PD-1 followed by sv6D (Group 3 in **a**), red; PBS, black

Intraperitoneal injection of a monoclonal anti-mouse PD-1 is modestly effective in mice with ovarian cancer model [82, 83]. We tested whether sv6D would extend effectiveness of anti-PD-1. Five doses of anti-PD-1 (200 μg in PBS) were injected intraperitoneally on alternate days between days 41 and 49. sv6D was injected subcutaneously immediately after the treatment with anti-PD-1 and continued on alternate days to the end of the study (Fig. 7a, group 3). When administration of sv6D to a group that had been treated with anti-PD-1 was delayed until day 87, after weight of the animals began to increase (group 5), essentially complete suppression of ascites formation was sustained for several weeks. Group 6, with a hiatus of one week after a week of treatment, survived only slightly longer than animals treated with PBS. This observation appears similar to the unresponsiveness of T cells that was observed in mice within a week after treatment with antibodies against C-type lectin receptors on DCs [12, 84]. For comparison, survival of animals with continuous alternate-day injection of sv6D alone, from the experiment shown in Fig. 6c, is included as group 7. The median survival of control groups treated with PBS in both experiments was the same (122 days). Kaplan-Meier survival curves are shown in Fig. 7b.

### Toxicity of peptide treatment

Although weight gain is a good measure of disease progression in this model of disseminated ovarian cancer in the peritoneal cavity, consideration of mean group weight is complex. Because this is an intact biological model, inherent variation exists. As disease in individual animals within a treatment group progress, the variation in weight within the treatment group increases. When an individual animal reaches end-stage disease and is euthanized, the mean group weight may drop and the variation within the group decreases. Given this complexity, there remained several instances in which reduced progression of disease within a treatment group was reflected in differences in weight that reached statistical significance.

**Fig. 8:**
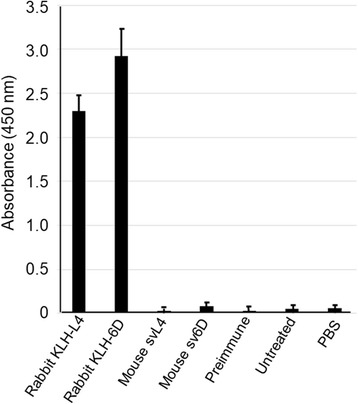
Test for antigenicity of svL4 and sv6D. Anti-sera were generated in rabbits against KLH-conjugates of the sequences of svL4 or sv6D and diluted 1:10. Mouse sera were collected after alternate-day injections of svL4 over 3 months, diluted 1:1 with PBS and added to protein-A/G-coated wells. Biotinylated svL4 or sv6D was added to wells and bound peptide was detected with a streptavidin-peroxidase conjugate. The figure includes values ± S.D. for sera from 8 treated mice in each group assayed separately

It is important to note that during and after drug administration there was no change in mouse behavior, indicating no overt toxicity related to peptide treatment. Further, repeated injection of peptide in the same region resulted in no apparent irritation or formation of fibrous or granulomatous tissue. Attempts to detect antibodies that bind the peptides in sera from mice injected on alternate days for 3 months with 1 nmole/g svL4 or sv6D were negative (Fig. 8), which indicated that the peptides are not antigenic in mice.

To determine whether the peptides induced a significant release of cytotoxic cytokines, sera were collected from female Balb/c mice into which breast cancer 4 T1 cells had been implanted. After the tumors had grown over a period of 10 to 12 days to a size of ~ 500 mm^3^, svL4 was injected subcutaneously at doses of 0.1 or 1.0 nmole/g. Sera were prepared 4 h after injection and analyzed with an array of 308 cytokines/chemokines (see Additional file 3: Figure S3). Ratios of the amounts of selected cytokines from treated vs. untreated mice are listed in Table 3.

**Table 3:** Ratios of the levels of selected cytokines in sera from treated vs. untreated animals

Cytokine	Tumor-bearing	Expression	Healthy
IL-1α	2.7	Activated Macrophages	0.86
IL-12p70	3.5	Dendritic cells and macrophages	No change
IL-16	4	Released by lymphocytes	0.57
IL-27	4.5	Antigen-presenting cells, promotes Th1 responses	0.55
IL-28	4.3	Augments IFN-γ release, cytotoxic potential of CD8^+^ T cells	High*
IL-31	3.5	Activated Th2 T cells	High*
MIP-2/CXCL2	~ 6	Secreted by monocytes and macrophages	No change
Pentraxin3	~ 4	Mononuclear phagocytes, dendritic cells and neutrophils	0.21
SPARC	~ 5	Osteoblasts, macrophages at site of wound repair	No change
TIMP-2	3	Metastasis suppressor, expressed by monocytes, placenta	0.45
TNFα	45*	Activated monocytes and macrophages	0.21
TLR2	4.2	Activated monocytes, DCs, macrophages, B and T cells	0.37
Lymphotoxin-α	12.5*	Produced by Th1 T cells	0.74
sHVEM	~ 2	Activated monocytes and lymphocytes	10
IFN-γ	High^*^	Activated lymphocytes	No change
CCL1	2.5	Activated T cells	No change
Lymphotactin	~ 3	Activated CD8^+^ cells	2.6

Markers of lymphocyte and monocyte activation in response to 0.1 nmole/g svL4 in Balb/c mice bearing breast tumors are compared with healthy mice. Sera from three animals were pooled for analysis with an array of 308 soluble factors. (*In these samples, the control value was negligible or low, thus the fold increase for treated samples appears high)

Several cytokines had negligible values in untreated mice, and thus the ratio of treated (although low) vs. untreated animals had a high value. Ratios of treated vs. untreated samples obtained in a separate experiment with healthy Balb/c mice are shown in the right-hand column of Table 3 for comparison. The most significant increase in the serum of healthy mice treated with the peptide was in the amount of soluble HVEM (Herpes virus entry mediator, also designated TNFRSF14), which was a prominent protein in serum and increased approximately 10-fold within 4 h after treatment. In the tumor-bearing mice, the level of soluble HVEM in treated animals was similar to that in treated healthy mice, but the level in untreated animals was higher than that in healthy control mice. Interestingly, cytokines that were strongly elevated in mice bearing tumors were often reduced in healthy mice in response to svL4 or showed no change. The amounts of inflammatory cytokines in the sera did not appear to reach toxic levels.

## Discussion

Peptides are uniquely suited to immunotherapy. Peptides are flexible in design, easily synthesized on a large scale, water soluble, and relatively stable. Although their use as vaccines has a long and successful history, peptides have also been designed that bind selectively to lectin-type receptors with high avidity ([85–87] and references therein). Their use in receptor-mediated immunotherapy is based on their ability to bind to regulatory, lectin-type, cell-surface receptors expressed by cells of the immune system, with avidities that are orders-of-magnitude greater than endogenous ligands. The interaction of multivalent ligands with multimeric receptors leads to dramatic increases in binding avidity, which decrease the concentration for half-maximal binding to the low nanomolar range. Therefore, peptides such as svL4 and sv6D, described in this report as mimetics of GalNAc, provide a means to modulate activity of immune cells. As shown in Fig. 5, the peptides promote a broad expansion of immune cell populations in the peritoneal cavity. Furthermore, long-term treatment did not induce production of antibodies in the mouse (Fig. 8).

To explore whether targeting C-type (Ca^2+^-dependent) lectin-type receptors offers a novel immunotherapeutic approach to cancer treatment, our research focused on CLEC10A (CD301) that is expressed by several cell types at strategic stages in the immune pathway, whose activation can lead to immune responses and a good therapeutic outcome [12]. CLEC10A expression is upregulated during differentiation of myeloid committed progenitor cells [88]. Possibly, peptide binding to these cells induces proliferation of cells that differentiate into mature, activated immune cells in the peritoneal cavity (see Additional file 4: Supplemental Material). Our data support the hypothesis that the peptides svL4 and sv6D engage CLEC10A on dermal DCs and immature peripheral DCs and promote cellular maturation. Interaction of T cells with mature DCs within lymphoid tissues leads to their activation. DCs are integral to regulation of the immune system through multi-directional and reciprocal cross-talk, which leads to activation of other types of cells such as NK cells to support innate immunity [89, 90].

Alternatively-activated, tumor-associated macrophages, in particular the classical immunosuppressive M2a type, express CLEC10A [91]. Activation of the phagocytic activity of macrophages leads to reduction of T_REG_ cells in melanoma tumors [92, 93]. Preliminary results showed that treatment of melanoma-bearing mice with svL4 caused a dramatic reduction in T_REG_ cells within tumors (data not shown), which possibly resulted from nudging M2a cells to the more phagocytic M2b macrophages by engaging CLEC10A. Of importance for this study, CLEC10A is expressed by DCs in skin and peripheral immature DCs. Ligand binding to the receptor initiates maturation and migration of DCs to lymphoid tissues, where they engage in antigen presentation to T cells [34–36]. Targeting MGL2 on dermal DCs with a multivalent Tn antigen induced a potent MHC II-restricted T cell response and release of Th2 cytokines such as IL-4, IL-5, IL-10 and IL-13 in Balb/c mice [24].

An analysis of lectin-type receptors on human blood DCs and the extent of binding of an array of glycans revealed that prominent among the glycans that bound were those with terminal GalNAc residues [94]. This study by Rapoport et al. was performed to evaluate optimal vectors for delivering vaccines to DCs and concluded from these results that MGL (CLEC10A) is a promising target. However, IC_50_ for binding glycan-PAA conjugates was about 20 μM [94]. Multivalent peptides such as sv6D achieve an optimal cellular response with concentrations near 10 nM (Fig. 4) and preliminary data show that sv6D effectively activates T cells against an antigen in vivo (data not shown)*.*

The highly glycosylated, cell membrane protein CD45, which bears the Tn structure at positions 137 and 140 in exon B of the sequence [22], was identified as an endogenous ligand of CLEC10A/MGL [95]. Although the Tn antigen binds to the receptor with much lower avidity than the peptides svL4 and sv6D [32], and the affinity of an intact glycoprotein bearing a single sugar (K_I_ ≈ 23 μM [96]) is several orders of magnitude less than that of the peptides, these structures are conceivably competitive inhibitors. CD45 is expressed as several isoforms, with the full length protein containing exons A, B and C at the extracellular, variable region [97, 98]. Binding of CLEC10A to exon B-containing isoforms causes attenuation of T cell activity, apoptosis and immunosuppression [95]. However, activation of T cells leads to expression of shorter isoforms of CD45 such as CD45RO and CD45RA that lack exon B [99–102]. Moreover, maturation of DCs leads to down-regulation of CLEC10A [95], which would minimize inhibition by CD45 of T cell activation during treatment with the peptides.

Paclitaxel is currently used as a chemotherapeutic drug that acts by stabilizing microtubules and arresting cells in the cell cycle at the G2/M boundary [80, 81]. Paclitaxel is often combined with a platinum-based drug [103, 104]. Patients treated with these drugs experience a significant level of toxicity. Our data suggest that sv6D would serve as an effective combination drug without adding toxicity. However, because sv6D induces proliferation of immune cells, initiation of treatment should perhaps occur after the cell cycle inhibitory action of paclitaxel has dissipated [105]. Patients with ovarian cancer have a 15% overall response rate during treatment with antibodies against the inhibitory receptor PD-1 (pembroliumab or nivolumab in human therapy) [106, 107]. The antibodies exhibit modest effectiveness in mouse models [82, 83]. The peptide sv6D inhibited accumulation of ascites to a greater extent than anti-PD-1 and also was effective in combination with the antibody. Particularly interesting is the effectiveness of sv6D when administration was delayed following anti-PD-1 (Fig. 7). Our peptide drugs satisfy the criteria described by Hamanishi et al. [108], who concluded that “particularly important in ovarian cancer, (which) is not associated with a high response rate, anti-cancer treatments are considered to be excellent if they are associated with low medical costs, low toxicity and high ‘benefits’ (anti-tumor response).”

## Conclusion

Our data support the hypothesis that GalNAc-specific lectins such as CLEC10A, expressed by DCs in the skin and immature DCs and macrophages, are target(s) for the peptides svL4 and sv6D. Extensive evidence supports initiation of a Ca^2+^ signal upon ligand-induced endocytosis of such Ca^2+^-dependent receptors [12]. Subsequent maturation of DCs leads to activation of T cells and other immune cells by cross-talk [90]. The result is a broad stimulation of the immune system that, although not antigen specific, is adaptable to various cancers and infectious diseases. sv6D possibly can be used as a monotherapy, and the lack of apparent toxicity also offers the potential that expansion of immune cell populations by the peptides may be a foundational treatment upon which combination immuno-therapies can be built. Although our data thus far support binding of svL4 and sv6D to lectins specific for GalNAc, it is possible that the peptides may also bind to other receptors not assayed in this study. The full extent of the activities of peptides svL4 and sv6D must await further research.

## Additional files

Additional file 1: **Figure S1.** Pseudocolor scatter plots of SSc vs. FSc from flow cytometric analyses of peritoneal cells from healthy (a) C57BL/6 or (b) Balb/c mice 24 h after injection with various doses of svL4. The population of small cells is circled. Graphical presentations of duplicate measurements of this population are expressed as a percent of total events. Peritoneal cells from 2 animals were pooled for each analysis. (TIFF 1300 kb)

Additional file 2: **Figure S2.** Increase in the population of small cells in the peritoneal cavity of Balb/c mice bearing tumors of breast 4T1 cancer cell line. svL4 (1 nmole/g) was injected on day 0 and day 2, with analysis 24 h after each injection. a) Pseudocolor scatter plots of peritoneal cells from untreated or treated mice on day 3, 24 h after the second injection. The low SSc and low FSc population is circled. b) The bar graph shows the low SSc and low FSc population presented as percent of total cells from analyses at days 1 and 3. Untreated animals, blue; treated animals, orange. Peritoneal cells from 3 animals were pooled and analyzed in triplicate. c) Histograms of normalized SSc vs. FSc for samples of peritoneal cells from Balb/c mice on day 3, from Fig. S2b, i.e., 24 h after the second injection of svL4. Blue trace, untreated animals; red trace, svL4-treated animals. (TIFF 1985 kb)

Additional file 3: **Figure S3.** Cytokines/chemokines in the sera of 4T1 tumor-bearing Balb/c mice treated with svL4 at doses of 0.1 nmole/g (orange) or 1 nmole/g (grey) body weight as compared with samples from animals injected with PBS (blue) 4 h after subcutaneous injections. Note: TNF-β is the same as lymphotoxin-α. Values indicate relative densities of dots on the mouse L-308 membrane array as analyzed by RayBiotech, Inc. (Norcross, GA). (PPTX 541 kb)

Additional file 4: Supplemental Material. (DOCX 28 kb)

## Abbreviations

ASGPR-1: asialoglycoprotein receptor-1
CLEC10A: calcium-dependent lectin-type receptor family member 10A
CRD: carbohydrate recognition domain
DCs: dendritic cells
DC-SIGN: dendritic-cell specific intercellular adhesion molecule (ICAM)-3 grabbing non-integrin
Gal: galactose
GalNAc: *N*-acetylgalactosamine
GalNAc-PAA: GalNAc conjugated to polyacrylamde
KLH: keyhole limpet hemocyanin
NQHTPR: Asparagine-Glutamine-Histidine-Threonine-Proline-Arginine
RMSD: root-mean-square deviation
S.D.: standard deviation
