# Supplementary figures and images for: An innovative immunotherapeutic strategy for ovarian cancer: CLEC10A and glycomimetic peptides
Source: J Immunother Cancer. 2018 Apr 17;6:28. doi: 10.1186/s40425-018-0339-5 (PMC5905120; doi:10.1186/s40425-018-0339-5)

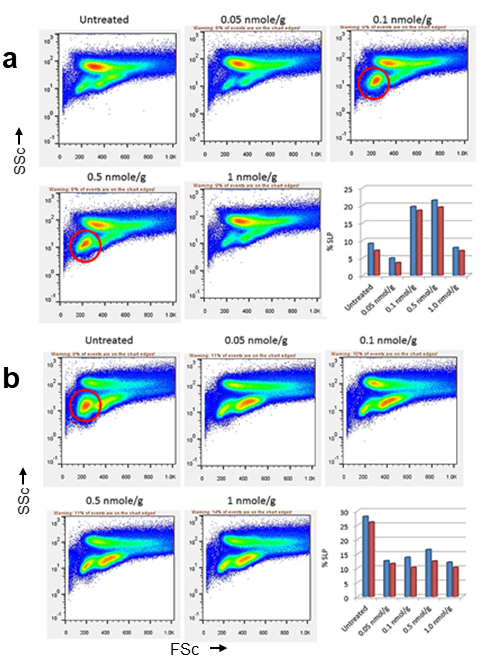

Supplement: Supplementary file 1 — Figure S1. Pseudocolor scatter plots of SSc vs. FSc from flow cytometric analyses of peritoneal cells from healthy (a) C57BL/6 or (b) Balb/c mice 24 h after injection with various doses of svL4. The population of small cells is circled. Graphical presentations of duplicate measurements of this population are expressed as a percent of total events. Peritoneal cells from 2 animals were pooled for each analysis. (TIFF 1300 kb) [file 40425_2018_339_MOESM1_ESM.tif]

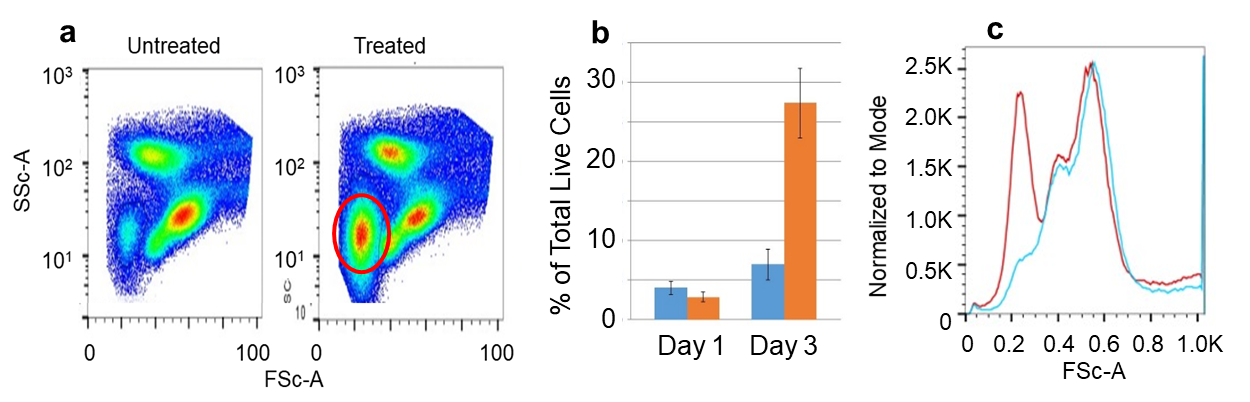

Supplement: Supplementary file 2 — Figure S2. Increase in the population of small cells in the peritoneal cavity of Balb/c mice bearing tumors of breast 4T1 cancer cell line. svL4 (1 nmole/g) was injected on day 0 and day 2, with analysis 24 h after each injection. a) Pseudocolor scatter plots of peritoneal cells from untreated or treated mice on day 3, 24 h after the second injection. The low SSc and low FSc population is circled. b) The bar graph shows the low SSc and low FSc population presented as percent of total cells from analyses at days 1 and 3. Untreated animals, blue; treated animals, orange. Peritoneal cells from 3 animals were pooled and analyzed in triplicate. c) Histograms of normalized SSc vs. FSc for samples of peritoneal cells from Balb/c mice on day 3, from Fig. S2b, i.e., 24 h after the second injection of svL4. Blue trace, untreated animals; red trace, svL4-treated animals. (TIFF 1985 kb) [file 40425_2018_339_MOESM2_ESM.tif]

## Slide 1
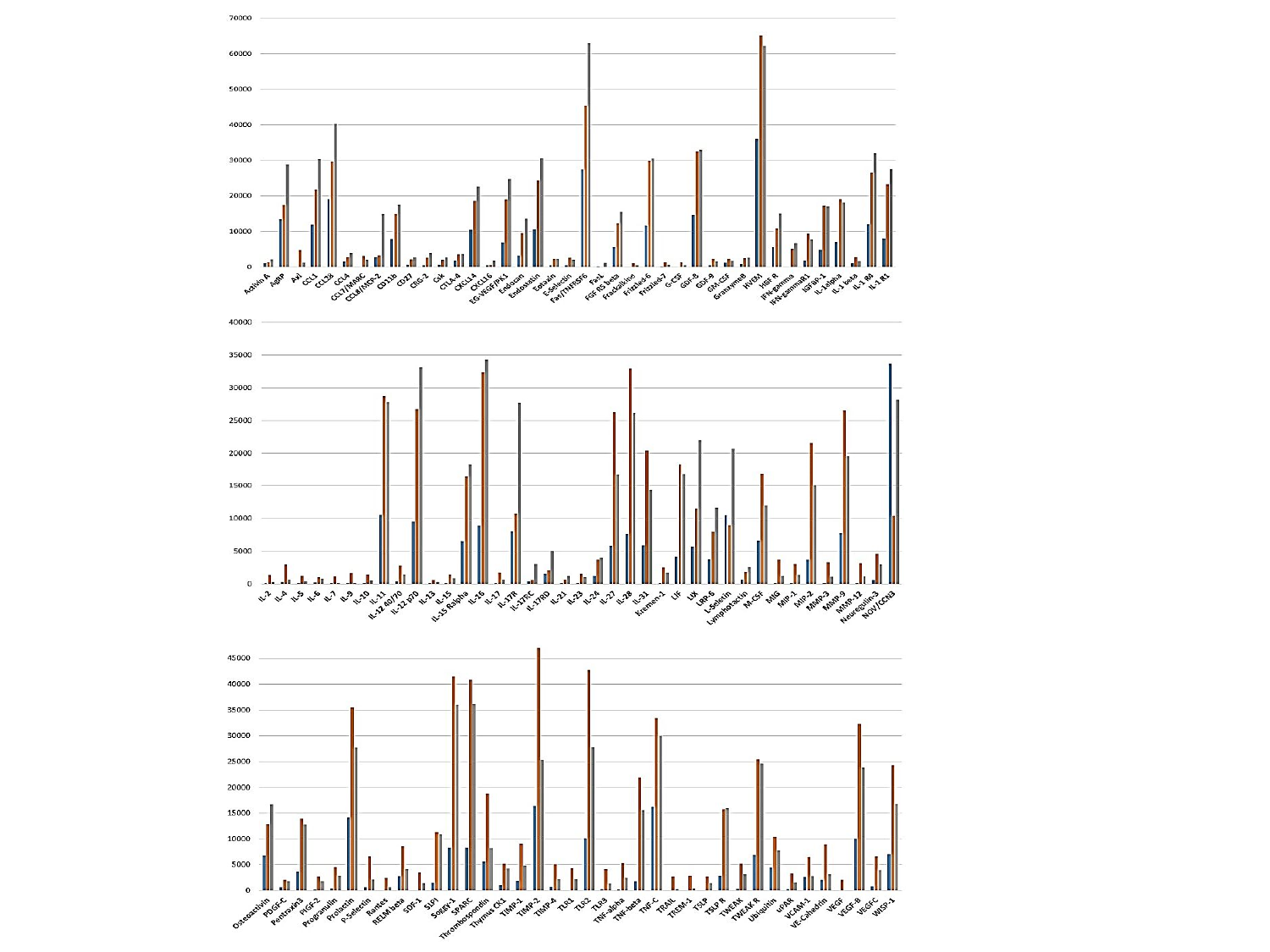

Supplement: Supplementary file 3 — Figure S3. Cytokines/chemokines in the sera of 4T1 tumor-bearing Balb/c mice treated with svL4 at doses of 0.1 nmole/g (orange) or 1 nmole/g (grey) body weight as compared with samples from animals injected with PBS (blue) 4 h after subcutaneous injections. Note: TNF-β is the same as lymphotoxin-α. Values indicate relative densities of dots on the mouse L-308 membrane array as analyzed by RayBiotech, Inc. (Norcross, GA). (PPTX 541 kb) [file 40425_2018_339_MOESM3_ESM.pptx]
