# Supplementary material for: An innovative immunotherapeutic strategy for ovarian cancer: CLEC10A and glycomimetic peptides
Source: J Immunother Cancer. 2018 Apr 17;6:28. doi: 10.1186/s40425-018-0339-5 (PMC5905120; doi:10.1186/s40425-018-0339-5)
Supplement: Supplementary file 4 — Supplemental Material. (DOCX 28 kb) [file 40425_2018_339_MOESM4_ESM.docx]

**Supplemental Material**

**An Innovative Immunotherapeutic Strategy for Ovarian Cancer:**

**CLEC10A and Glycomimetic Peptides**

Laura L. Eggink^1^, Katherine F. Roby^2^, Robert Cote^1^ and J. Kenneth Hoober^1*^

**Studies on a population of small peritoneal cells**

An injection of peptide svL4 into C57BL/6 mice resulted—within 24 h, in a several-fold increase in a population of cells in the peritoneal cavity that appear as small in volume (low end of FSc axis) while also containing minimal intracellular complexity (low end of SSc axis) in a flow cytometric scatter plot (circled in Fig. S1**a**). Essentially no increase was observed in mature immune cells within this time period (data not shown). Significant changes in populations of mature immune cells in C57BL/6 mice were not observed until after the second, alternate-day injection of the peptide (Fig. 5). In Balb/c mice, the low FSc and low SSc population was abundant but decreased to a minimal level 24 h after an injection of svL4 (Fig. S1**b**). Concomitant with the decrease in this population in Balb/c mice was an approximately 2-fold increase in mature immune cells within 24 h after a single injection of svL4 (data not shown).

Because of the dynamic and reciprocal correlation with mature immune cells, attempts were made to identify this population of small cells. They did not stain significantly with previously used identification markers. Cells were then stained with antibodies against the markers Lin (lineage-associated markers for lymphocytes and monocytes), CD90.2 (Thy-1, a T cell marker), CD34 (a hematopoietic progenitor cell marker) and CD117 (stem cell growth factor receptor, also designated c-Kit, expressed highly on common myeloid progenitor cells but at low levels on lymphoid progenitor cells). In untreated C57BL/6 mice, when the number of these small cells in 4 of 5 animals was low, most were Lin^+^; in 1 of 5 animals the population was larger and 23% were Lin^+^. Approximately 35% overall were CD90.2^+^. In C57BL/6 mice treated with 0.05 nmole/g svL4, this population decreased further (Fig. S1**a**) and the average percentage of Lin^+^ cells increased slightly, with the percentage in individual animals ranging from 31% of the population to 84%, in an inverse pattern with the size of the population. The CD90.2^+^ cells decreased more than 50% as this population increased. In C57BL/6 mice injected with 0.1 or 0.5 nmole/g svL4 (Fig. S1**a**) and untreated Balb/c mice, when this population was large (Fig. S1**b**), nearly all cells were Lin^Neg^ and CD90.2^Neg^. After treatment with svL4, when the size of this population in Balb/c mice had decreased, most cells were Lin^+^ (from 51% to 72%), and about 15% were CD90.2^+^. In all animals, when this presumed undifferentiated population was small, most remaining cells were Lin^+^. When this population was large, most cells were Lin^Neg^. Stem and progenitor cells are Lin^Neg^CD90.2^Neg^, but these peritoneal cells also lacked CD34 and CD117. Whereas human progenitor cells are CD34^+^, mouse hematopoietic stem cells are CD34^low^ or CD34^Neg^. Thus, although likely a mixture of different cell types, when this population is large the majority appear to be progenitor cells.

Another experiment was performed in which 6 to 8 week-old female Balb/c mice were anesthetized with isoflurane and inoculated with 5 x 10^5^ 4T1 breast cancer cells in the 4^th^ mammary fat pad. After tumors had reached a volume of at least 500 mm^3^, the peritoneal population of low FSc and low SSc cells was minimal (Fig. S2**a**). Twenty-four hours after an injection of svL4, the size of this population was not changed significantly. However, a dramatic increase in this population occurred within 24 h after a second injection (Figs. S2**a** and S2**b**). The analysis indicated about 3% of this population was CD19^+^ and 0.5% was CD3^+^, but the cells did not stain with other identifying markers. Shown in Fig. S2**c** are histograms from an analysis of peritoneal cells from a treated animal at day 3, *i.e*., 24 h after the second injection of 1 nmole/g svL4 (red trace), as compared with cells from an untreated animal (blue trace), which revealed a dramatic increase in the population of small cells. We conclude that this population is possibly progenitor cells and that additional treatments would lead to maturation of this population, as observed with healthy animals. The overall histograms in Fig. S2**c** were not significantly different between cells from untreated and treated animals, except for the low FSc population, which was highly responsive to treatment with the peptide.

**Analysis of changes in cytokine/chemokine levels in Balb/c mice after injection with svL4.**

For analysis of a cytokine response *in vivo* to subcutaneous injection of peptide, the Balb/c mice with implanted 4T1 breast tumors (described above) were randomized into groups of 3 and dosed with either 0.1 or 1.0 nmole/g of svL4. At 4 h post dose, terminal blood was pooled from 3 animals per group and prepared for serum. Shown in Fig. S3 are the relative levels of cytokines/chemokines in sera in response to injections of 0.1 (orange bars) or 1.0 nmole/g (grey bars) svL4 as compared with animals injected with PBS (blue).

**Supplemental Figure Legends**

**Fig. S1.** Pseudocolor scatter plots of SSc vs. FSc from flow cytometric analyses of peritoneal cells from healthy (**a**) C57BL/6 or (**b**) Balb/c mice 24 h after injection with various doses of svL4. The population of small cells is circled. Graphical presentations of duplicate measurements of this population are expressed as a percent of total events. Peritoneal cells from 2 animals were pooled for each analysis.

**Fig. S2**. Increase in the population of small cells in the peritoneal cavity of Balb/c mice bearing tumors of breast 4T1 cancer cell line. svL4 (1 nmole/g) was injected on day 0 and day 2, with analysis 24 h after each injection. **a**) Pseudocolor scatter plots of peritoneal cells from untreated or treated mice on day 3, 24 h after the second injection. The low SSc and low FSc population is circled. **b**) The bar graph shows the low SSc and low FSc population presented as percent of total cells from analyses at days 1 and 3. Untreated animals, blue; treated animals, orange. Peritoneal cells from 3 animals were pooled and analyzed in triplicate. **c**) Histograms of normalized SSc vs. FSc for samples of peritoneal cells from Balb/c mice on day 3, from Fig. S2**b**, *i.e*., 24 h after the second injection of svL4. Blue trace, untreated animals; red trace, svL4-treated animals.

**Fig. S3.** Cytokines/chemokines in the sera of 4T1 tumor-bearing Balb/c mice treated with svL4 at doses of 0.1 nmole/g (orange) or 1 nmole/g (grey) body weight as compared with samples from animals injected with PBS (blue) 4 h after subcutaneous injections. Note: TNF-β is the same as lymphotoxin-α. Values indicate relative densities of dots on the mouse L-308 membrane array as analyzed by RayBiotech, Inc. (Norcross, GA).
